# Tonic non-colonic convulsions-status epilepticus- as the presenting complaint of COVID-19

**DOI:** 10.1016/j.amsu.2022.103744

**Published:** 2022-05-10

**Authors:** Omar Al Jandale, Mohammad Badr Almoshantaf, Aya qaddah, Agyad Bakkour, Eman Mohammed sharif Ahmed

**Affiliations:** a Department of Cardiology, Damascus University, Damascus, Syria; b Department of Neurosurgery, Ibn Al-Nafees Hospital, Damascus, Syria; c Department of Neurology, Damascus University, Damascus, Syria; d Faculty of Medicine, Albaath University, Homs, Syria; e NILE VALLY UNIVERSITY, Atbra, Sudan

**Keywords:** COVID-19, Coronavirus, Epilepsy, Seizure

## Abstract

**Introduction:**

COVID19 might present as neurological symptoms including headaches, dizziness, anosmia, stroke, and loss of consciousness. Cases with severe COVID-19 are more likely to be complicated by neurological symptoms, but tonic non-colonic convulsion is still a rare presentation of COVID19 as an initial symptom.

**Case presentation:**

A 23-years old male presented to the ambulance with a complaint of loss of consciousness for more than 1 h and tonic convulsions without clonic movements. The investigations including computed tomography for the brain and chest and lumbar puncture were within normal range and the diagnostic workup concluded that COVID-19 is the cause of the status epilepticus.

**Discussion:**

Our case demonstrates a tonic non-clonic convulsion as a possible complication for COVID-19 infection as some respiratory viruses can cause neurological symptoms. After excluding the co-incidence of other pathological etiologies, we highly suspect that the seizures in our case are generated by COVID-19 infection.

**Conclusion:**

This case represents a rare case in the literature review which can increase the awareness of tonic non-clonic seizures and other neurological manifestations as the presenting symptom of the COVID19.

## Highlights

• COVID-19 can manifest with neurological symptoms.
• Severe cases of COVID-19 are more likely to be complicated be rarer symptoms.
• Tonic non-Clonic seizures are rare in COVID-19 and have never been reported before.

## 1 Introduction

Neuro-invasion related complications are widely documented in most human coronaviruses (OC-43, 229E, MERS and SARS). Headaches, dizziness, and anosmia are all part of the neurological spectrum of possible symptoms. Less commonly, some cases of encephalopathy, encephalitis, necrotizing hemorrhagic encephalopathy, stroke, epileptic seizures, rhabdomyolysis and Guillain-Barre syndrome, were also reported to be associated with SARS-CoV-2 infection [1].

The incidence of neurological complications due to SARS-CoV-2 is still unknown. However, reports have mentioned that cases with severe COVID-19 are more likely to be complicated by neurological symptoms [2]. New-onset seizures is a possible complication in critically ill patients with COVID-19. Such seizures should be considered acute symptomatic seizures and its etiology must be determined and managed accordingly as fast as possible. Extensive clinical, neurological, radiological, and electrophysiological investigations are necessary to clarify the role of COVID-19 in causing in these patients. It's worth mentioning that attempts to isolate SARS-CoV-2 from CSF could have some value in treatment plan [3].

The literature does not provide enough evidence for seizure worsening during the course of a SARS-CoV-2 infection. But it's theocratized that the seizures may be triggered by COVID-19 associated fever. Additionally, advanced stages of COVID-19 infection can result in hypoxic encephalopathy and cytokine storm, which can develop acute seizures [4].

Despite the low number of reported cases of seizures in COVID-19, most reported seizures are the tonic-clonic type. We experienced a case of tonic non-clonic convulsions in a young man with a remarkable history. We are reporting a rare neurological manifestation of COVID-19 which can widen the spectrum of possible types of seizures complicating COVID-19.

## 2 Case presentation

A 23-years old male presented to the emergency department with a complaints of a loss of consciousness associated with tonic convulsions, passing of urine and tongue biting. His medical history only included bacterial meningitis at 6-years of age without any mention of seizures or neurological presentation. There was no another abnormality in his medical history.

During the episode, Glasgow coma scale (GCS) at admission was 6/15. His blood pressure was 130/80, temperature was 37.2, and his pulse was 86/min. The seizure episode was stopped after intravenous infusion 5mg of midazolam. After that, initial neurological examination was normal except the existence of positive bilateral plantar reflexes. According to his parents, he was completely normal before the episode and only complained of fatigue.

His biochemical tests including cell blood count (CBC), glucose, urea and electrolytes were all within normal range except for lymphopenia (15%-1000/mm^3^).

After admitting him to the neurology department, chest computed tomography was ordered, which was within normal limits without any noticeable infiltration or opacities. However, as both government and international guidelines advice, two nasopharyngeal swabs were performed and were found to be positive for SARS-CoV-2 on qRT-PCR. As a result, he was given acyclovir, phenytoin, ceftriaxone, vancomycin, and dexamethasone as an empiric therapy for a suspected bacterial meningitis and status epilepticus. Before antibiotics were given, lumbar puncture was performed and the results were as follows: Turbid before sedimentation, Clear after sedimentation, Leukocytes 20/mm^3^, red cells 10/mm^3^, Protein 62.2 mg/dl, Glucose 74 mg/dl, LDH 15. PCR test for HSV came back negative.

**Fig. 1:**
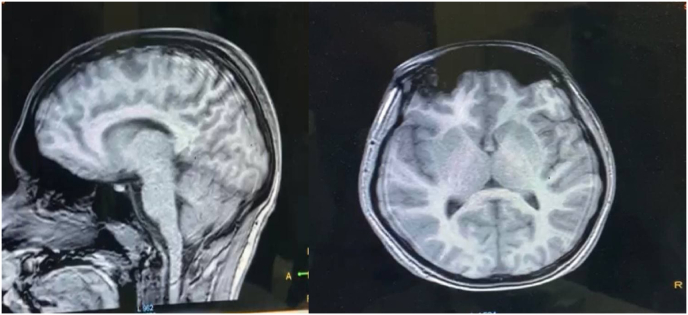
[a, b]: MRI revealed normal finding at First time(1T).

After half of hour, he returned to the normal status with a GCS of 15. Later, brain magnetic resonance imaging (MRI) with contrast revealed no neurological abnormalities (Fig. 1[a, b]). The patient was discharged in the same days and his status was improved without any complications during the following up in an outpatientsetting for one month.

## 3 Discussion

Our case demonstrates a tonic non-clonic convulsion as a possible complication for COVID-19 infection. Unlike common knowledge, some respiratory viruses such as the human coronavirus (SARS-CoV-2), can infect other organ systems than respiratory system, including the central nervous system (CNS). Clinical manifestation of CNS infection includes acute encephalitis with either febrile or afebrile seizures, status epilepticus, and chronic encephalopathies. As, coronaviruses share the same infection pathway, understandably, a question of whether SARS-CoV-2 possess the same neuro-invasion features as SARS-CoV had to be asked [4]. As a result, there are growing evidences that shows that neurotropism is a shared feature among coronaviruses subtypes.

Naturally, the possibility of the existence of another cause of these seizures still stands. However, the patient was manifested for the first time without any previous family history. Moreover, blood tests and MRI were all negative. On clinical examination, we excluded common pathologies that are known for causing seizures. Meningitis was excluded as there were no fever, stiff neck, signs of photophobia, or headaches. A space-occupying lesion was excluded by the MRI. SARS-COV-2 was the most probable cause of the presentation and the patient was treated accordingly.

Mao et al., have studied the neurological manifestations of patients that have been confirmed to be COVID-19 positive in their retrospective study. Among the 214 patients they have studied, about 36.4% patients have developed neurological manifestations. The more severe was the infection, the likelihood of neurologic symptoms to appear increased. Neurologic symptoms included acute cerebrovascular, impaired consciousness, dizziness, headache, hypogeusia, hyposmia, and skeletal muscle injury. Only one patient (0.5%) has found to experience epilepsy as a result of a severe COVID-19 infection. According to this, it's clear that epilepsy is not a common complication to be observed routinely in COVID-19 patients [5].

In a case report by Fasano et al. [6], they described a similar case to ours of a motor seizure as a result of SARS-CoV-2 infection. Unlike our case's non-clonic epilepsy, their case demonstrated a tonic clonic seizure. Both cases reported no previous neurological signs which should raise more suspicion towards SARS-CoV-2 potential causality to a spectrum of epileptic disorders.

Another study by Anand et al., have studied retrospectively seizures in COVID-19 in terms of risk factors, clinical features, and outcomes. Throughout the study collection period of 45 days in a tertiary care center, only 7 COVID-19 positive patients presented with seizures. Three of those had a prior history of well-controlled epilepsy. In contrast to our case's non-clonic episode, five of 7 patients had generalized tonic clonic seizures and two of them had focal seizures. Interestingly, similar to our case, all patients presented to the emergency department because of the ongoing episode [7]. This nearly eliminates the probability of hospital-acquired pathology being a cause for such manifestations.

In conclusion, our case only suggests a possible correlation between seizures disorders new onsets and SARS-CoV-2 infection. We advise that further attention should be given when treating COVID-19 patients that begin to show neurological symptoms. In addition, emergency departments’ respiratory teams should always be prepared to face epileptic episode when treating COVID-19 patients especially severe cases. We believe that more studies can determine if the association is only by chance or causative.

## Ethical approval

N/A.

## Sources of funding

This research did not receive any specific grant from funding agencies in the public, commercial, or not-for-profit sectors.

## Author contribution

All authors have participated in writing and reviewing the manuscript.

## Registration of research studies

Not applicable.

## Guarantor

Mohammad Badr Almoshantaf.

## Consent

N/A.

## Ethical approval

This case report didn't require review by ethics committee, Aleppo university hospital, Aleppo university, Aleppo-Syria.

## Consent for publication

Written informed consent was obtained from the patient for publication of this case report and accompanying images. A copy of the written consent is available for review by the Editor-in-Chief of this journal on request.

## Provenance and peer review

Not commissioned, externally peer reviewed.

## Declaration of competing interest

All authors declare no conflict of interest.
